# Hospital Worthies

**Published:** 1886-10-09

**Authors:** Samuel Morley


### Hospital Worthies.

### THE LATE MR. SAMUEL MORLEY.

"How far that little candle throws its beams!
So shines a good deed in a naughty world."

**Princely Munificence.** These words of the prince of dramatists come uppermost to the mind when we think of that light which, not long since, was shedding, as it had shed for years, its beams far and wide around. Posterity will remember Samuel Morley, not as a successful London merchant, not as a politician, not as a leader of Nonconformity and thought, but as one of the greatest philanthropists of the age. How many amongst the wealthy of to-day spend £20,000 a year upon religious and benevolent work yet, great as this sum is, it is declared to be below the aggregate of the calls which annually fell on Morley's purse.

**His Hospital Work.** The daily press have already rendered a noble tribute to the memory of this large-hearted man, but there is one matter, in which we are more immediately concerned, to which we desire to direct attention. We refer to Mr. Morley's connection with those excellent movements —the Hospital Sunday and Saturday Funds. To the work of these funds Mr. Morley brought the weight of his influence, and the assistance of his speech and presence for many years. Mr. Morley recognised that the hospitals of London, dependent as they are to the extent of some £400,000 a year upon voluntary sources, needed and deserved the advocacy and the work of the best of her citizens, and he never failed to respond willingly to any call which their necessities made upon his time and purse. But what was even more valuable still, he was prepared to, and did devote much time to the advocacy of their claims, and to the development of organisations which aimed at increasing the pecuniary support of hospitals by arousing the sympathy of the public at large.

**Hospital Sunday.** Mr. Morley was present at the first meeting at the Mansion House, some fifteen years ago, when it was decided to establish a Metropolitan Hospital Fund. From that day to the day of his death he was a member of the Council, from the meetings of which, for many years, he was seldom or never absent. His speeches, in the early history of the Fund, materially contributed to its extension and success. In later years he became a member of the Distribution Committee of the Fund, the responsibilities of which are very onerous. The fact that the decisions of the Distribution Committee as to the disposal of the funds raised on Hospital Sunday have been accepted without dispute, speaks volumes for the impartiality and efficiency of this committee. Mr. Morley's death creates a vacancy which it will be difficult to fill, as he represented elements seldom combined in the same individual, and occupied an unique and important position in relation to the work of the Metropolitan Hospital Sunday Fund Organisation.

**Hospital Saturday.** Mr. Morley probably gave more time and personal attention to the Hospital Saturday Fund in London than he ever devoted to any other single charitable organisation. Those who can recall the condition of this fund eight years ago, and contrast it with that of today, will see how considerable, if perhaps gradual, has been the improvement accomplished during the presidency of Samuel Morley. It was in the year 1878, upon the resignation of Captain Mercier, that Mr. Frewer, the secretary, and Mr. Mackenzie waited upon Mr. Morley for the purpose of inviting him to accept the position of President. At first Mr. Morley hesitated. He wanted to know why his name had been selected, and suggested that there were others more fitted for the position than himself. It was represented to him that the movement had, in all probability, a great future before it, and that the Council thought that his name would be of immense assistance to them in popularising the work among working people. "Well," said Mr. Morley, "I will accept office, if you think I shall be doing any good; I rely on your assurance that I shall."

**Mr. Morley's Presidency.** The movement during Mr. Morley's presidency, although not making as much progress as some of its supporters could have wished, has gradually expanded, the annual collection now being about double what it was when Mr. Morley first became associated with it. Looking back upon the state of things which existed eight years ago, the Hospital Saturday Fund appears now in a most creditable position, and the whole organisation has improved and developed since Mr. Frewer became secretary. Thus the receipts have increased from £6,141 in 1874 to £11,192 in 1885, whereas the expenses of collection have fallen from £6,056, or 35.2 per cent., the average expenditure of the first three years, to £5,053, or 14.5 per cent. during the three years ended 1885.

**His Work.** Mr. Morley made it a rule never to undertake a position in which he would not take an active and a personal interest; in other words, he would never lend his name and influence to a movement which might possibly be a bogus concern. Each of the annual meetings of the Hospital Saturday Fund found Mr. Morley in the chair, except the meeting of 1885, when Mr. Arnold Morley presided. On all occasions, when matters of importance came before the Council, Mr. Morley was sure to be in attendance.

**Mr. Morley Opposed.** One incident occurs to us, as so thoroughly characteristic of the man, that it is deserving of record here. In 1882 a proposal was made to establish a Seaside Convalescent Home at St. Margaret's Bay, for working-men in connection with the Hospital Saturday movement. The suggestion, which, we believe, originated with the Secretary, Mr. Frewer, was warmly received by the Council. Mr. Morley had, however, serious doubts as to whether the starting of such an institution came within the province of the movement, and at his wish a large meeting of the collectors of the fund was held at Exeter Hall, when the proposal was submitted for consideration. Mr. Morley, who took the chair, moved an amendment to the effect that the matter was rather one for the consideration of the friendly societies of London. The result, however, was a large majority favourable to the proposal. Mr. Morley thereupon declared his intention to support the undertaking! A few weeks afterwards Mr. Frewer called upon him to tell him how the matter stood, and to seek his co-operation and assistance. Mr. Morley at once said he would give £50 towards the establishment of the Convalescent Hospital. The conversation was continued, and the £50 was soon altered to £100. Mr. Morley went on to discuss with the Secretary the safeguards which it would be necessary to establish to insure the admission only of convalescents of the class intended to be benefited, the final result of the interview being, that the President drew a cheque for £200! The Home, which was opened in June 1883, has prospered, and already over one thousand patients have passed through it.

**Street Collections.** There was one opinion strongly held by Mr. Morley with reference to the Hospital Saturday movement which was opposed to the views of those taking a more active part in the work. He never cordially approved of the street collections, thinking that the sole aim of the organisation should be to secure the contributions of working men in their workshops and warehouses, especially remembering the importance of weekly or monthly contributions. Mr. Morley was undoubtedly right in this view, and had it prevailed we believe that the Metropolitan Hospital Saturday Fund would have reached £20,000 a year by this time. The Council of the Fund of course agreed with Mr. Morley as to the desirability of extending the workshop collections as far as possible, but they were of opinion that, for the present at least, the street collections, yielding as they did some £1,500 or £2,000, were an absolute necessity. Moreover, they held that ladies, habited in pretty dresses, when stationed in the leading thoroughfares all over London, afford at once an advertisement and an impetus to the movement which an indoor collection in workshop and warehouse does not possess. This view, however picturesque some may consider it, has been proved to be opposed to the best interests of the Hospital Saturday movement in large provincial towns. Few now doubt that it is desirable, in the interests of the fund in London, that the street collections shall be given up.

**His Loss.** Mr. Morley has passed away, and not only this vast city of four millions, but, indirectly, the whole Christian world, has lost a friend and benefactor; a man honest, upright, and good; a man whose glory was to further every good work; a man who, though a staunch sectarian, placed religion before religious sect. The Hospital Funds have, like many other philanthropic movements, suffered a severe loss by the death of a supporter whom it will indeed be hard to replace. Mr. Morley enjoyed considerable wealth, and used it generously, benevolently, and well.

